# Chelating Agents in Soil Remediation: A New Method for a Pragmatic Choice of the Right Chelator

**DOI:** 10.3389/fchem.2020.597400

**Published:** 2020-11-02

**Authors:** Valeria Marina Nurchi, Rosita Cappai, Guido Crisponi, Gavino Sanna, Giancarla Alberti, Raffaela Biesuz, Sofia Gama

**Affiliations:** ^1^Dipartimento di Scienza della Vita e dell'Ambiente, University of Cagliari, Cittadella Universitaria, Monserrato, Italy; ^2^Dipartimento di Chimica e Farmacia, University of Sassari, Sassari, Italy; ^3^Dipartimento di Chimica, University of Pavia, Pavia, Italy; ^4^Department of Analytical Chemistry, Faculty of Chemistry, University of Białystok, Białystok, Poland

**Keywords:** chelating agents, speciation, soil remediation, metal pollution, stability constants

## Abstract

Soil pollution by metal ions constitutes one of the most significant environmental problems in the world, being the ecosystems of extended areas wholly compromised. The remediation of soils is an impelling necessity, and different methodologies are used and studied for reaching this goal. Among them, the application of chelating agents is one of the most promising since it could allow the removal of metal ions while preserving the most meaningful properties of the original soils. The research in this field requires the joined contribute of different expertise spanning from biology to chemistry. In this work, we propose a parsimonious and pragmatic approach for screening among a range of potential chelating agents. This methodology, the *Nurchi's method*, is based on an extension of the Reilley procedure for EDTA titrations. This allows forecasting the binding ability of chelating agents toward the target polluting metal ions and those typically found in soils, based on the knowledge of the related protonation and complex formation constants. The method is thoroughly developed, and then tested by application to some representative cases. Its use and relevance in biomedical and industrial applications is also discussed.

## Introduction

Several metal ions, throughout the evolution of living organisms, resulted in essential for plants, animals, and man. Nevertheless, the introduction of exogenous metal ions that compete with essential ones can perturb the homeostasis of ecosystems until their disruption. For thousands of years, man used metals for necessities and progress, without forecasting about drawbacks of such custom. As result, the environment, as well as the human body, get intoxicated by polluting metal ions affecting water systems, plant, and animal life, mostly the whole ecosystem. The leading causes of metal environmental pollution are industrial drains, mining drains, urban wastes, residues of coal combustion, acid rains, fertilizers, and pesticides (Teng et al., 2020). Contamination by radionuclides is also a rising problem due to the increasing nuclear activities worldwide.

The metal pollution of water and soil compartments involves a definite and continuous exchange between them. Contrarily to soil pollution by organic substances, which can be transformed in the time into non-polluting ones by the degrading action of microbes, metal ions cannot be significantly transformed and persist indefinitely in the soil, or they pass to aquatic systems by the action of weathering. US Agency for Toxic Substances and Disease Registry periodically publishes a list of organic and inorganic toxic substances, ordered according to their inherent toxicity and to their dissemination, i.e., to their potential environmental impact. In 2019, arsenic, lead and mercury occupied the first three positions, being cadmium the seventh (ATSDR, 2020). These elements, commonly reported as heavy metals (Duffus, 2002), share the properties of being not biodegradable and of accumulating in living organisms and soils, entering the food chain, being its limits of concentration on soils well-defined (see details on following section), due to their toxicity.

Noxious elements exert their action through different mechanisms, such as absorption by plants entering the food chain directly or animal mediated, and hindrance of the microbial action in the soil. Consequently, heavily contaminated metal-polluted areas have led to definite health problems in the surrounding populations, whose intensity depends both on the kind of pollutant and on its concentration. In this way, the soils contaminated with toxic metal ions and radionuclides, constitute a significant problem both in advanced and in developing countries.

Due to the serious environmental and health consequences, with related social costs, the release of these metal ions has to be avoided—in the last 30 years different countries and supranational organizations adopted drastic resolutions to limit most of their uses–and efficient methods for their scavenging and removal from wastewaters and soils must be adopted. As such, prevention and remediation of polluted soils thus constitute important environmental, health and economic objectives that must be urgently faced by early aware local administrators. Several methods are being exploited, being *absorption* and (*bio) remediation* preferred due to their effectiveness and low cost.

The *remediation* methods can be roughly classified in two categories: (a) those that leave the toxic elements on the soil, immobilizing them to avoid their migration, and (b) those that remove contaminants from the soil, potentially saving it for future uses. In the present work, we are interested in the second class, and in particular in the *soil washing technology*. Among the different approaches on soil remediation processes, the *soil washing method* has attracted considerable attention, mainly for its ability to permanently remove heavy metals form the contaminated soil, combined with short duration procedure and remarkable cost effectiveness, when compared with other methods. Another advantage is the possibility of recovery of recyclable material or even energy production (Wuana et al., 2010; Cheng et al., 2020). The details of soil washing were primarily explored in the thorough review by Peters (Peters, 1999), being the *soil washing* described as a process in which excavated soil is first treated by physical separation, and it is then washed to remove contaminants using a chemical extracting solution. After the chemical treatments, the cleaned soil is returned to the original place. Due to this, the selection of effective and harmless washing reagents should be carefully pondered. Considering the methodology and the principle of the washing soil method, it is only applicable if there is an efficient transfer of the metal contaminants from the soil to the extracting solution. To achieve that, and due to the fact that heavy metals in the soils occur predominantly in an absorbed state, strongly bound to soil particles, is fundamental the use of extractant agents optimized for the solubilization of the target metals. For this purpose, several chemicals have been used, namely surfactants, cyclodextrins, organic acids and chelating agents. Among them, the selection and applicability is being studied on a case-by-case basis, as it depends on several parameters as the metal to remove and the characteristics of the soil as, for example, the pH. Furthermore, being one of the main goals of soil remediation the preservation as much as possible of the natural properties of the soil, it limits the number of possible extracting agents as, for example; strong acids can attack and degrade the soil structure, reason why the use of weak organic acids or chelating agents is often preferred (Wuana et al., 2010).

Chelating agents are applied in a considerable number of activities, spanning from medicine (Aaseth et al., 2016) to industry, from agriculture to domestic activities, from analytical chemistry to alimentary industry, and also in soil *remediation*, all due to their ability to complex metal ions. They act in different ways, such as (i) removing target metal ions from environment, (ii) avoiding metal precipitation, (iii) favoring ion crossing through biological membranes. Despite the fact that the complexing ability and the acid-base properties of chelating agents have been the object of extensive research, a detailed design of proper chelators according to the target metal ions and to their process requirements (solubility, lipo/hydrophilic properties, etc.) has not yet been fully exploited.

This work aims to give a methodological contribution in the choice of chelating agents for *soil washing remediation* based on our knowledge and expertise on the use of metal chelators in clinical and environmental applications (Crisponi et al., 1999, 2012; Villaescusa et al., 2002; Nurchi and Villaescusa, 2008, 2012; Nurchi et al., 2010, 2016; Crespo-Alonso et al., 2013; Aaseth et al., 2016).

In particular, a method that allow a preliminary screening among various potential ligands toward target metal ions will be proposed, based on simplifying assumptions, saving time and money need for a thorough experimental study on the behavior of these ligands in the field.

## Soil Concentration Ranges and Regulatory Guidelines for Relevant Metal Ions

A first step of the restoration of metal-polluted ecosystems requires a correct characterization of the state of pollution and preliminary knowledge of the characteristics of the soil. To access the concentration of metals contaminants in soil, total elemental analysis is usually the preferred method, as it is not affected by the chemical or physical form on which the metal is present. In this sense, the level of metal contamination is expressed by mg metal per kg of soil (mg Kg^−1^). This quantification is then the basis of the establishment of adequate remediation processes, and several guidelines can be recommended. This is done following two well-established parameters: intervention values and target values. Intervention values specify the concentration limits (in mg Kg^−1^ or mmol Kg^−1^) after which the quality of soil for human, animal and plant life starts to be severely compromised and concentrations in excess of the intervention values correspond to serious contamination. On the other hand, target values indicate the soil quality levels required for the full restoration of the functionality of soil. In Table 1 we report the intervention and target values for various metal ions of ascertained and potential toxicity, as previously reported by Wuana and Okieimen (2011).

**Table 1:** Soil concentration ranges, regulatory guidelines, intervention and target values for various metal ions of ascertained and potential toxicity.

**Metal ion**		**Intervention value**		**Target value**	
**Symbol**	**Atomic weight**	**mg.Kg^**−1**^**	mM^*^	**mg.Kg^**−1**^**	mM^*^
Hg	200.59	530	2.6	85	0.4
Cd	112.41	380	3.4	100	0.89
Pb	207.20	210	1.0	35	0.17
Cu	63.55	10	0.2	0.3	0.01
Ni	58.69	720	12.3	140	2.4

*Data from ref. Wuana and Okieimen (2011)*.

* *mM concentration corresponds to the concentration of a solution obtained suspending 1 Kg of soil in 1 L*.

## Method for Assessing the Chelating Ability

The right selection of the chelating agent is the fundamental step for a successful chelating agent-based soil washing process. With this in mind, we developed a simple method to assess the ability of different ligands in chelating metal ions, which provides at a glance information on this ability and allows a preliminary classification of the ligands on the base of their chelating properties toward the target metal ions. The procedure is based on an extension of Reilley's method used mainly in analytical applications of titrations with ethylene-diamine-tetraacetic acid (EDTA) (Reilley and Schmid, 1958; Crisponi et al., 2000).

Our method, from now on designated as *Nurchi's method*, is a simplistic method that focuses on the complex formation reaction between the metal ion M (assumed as a free ion in solution) and the chelating agent L, neglecting all the influences of non-thermodynamic contributions, including the binding with soil components. In a first stage, we assume (a) that the ligand does not interact with protons, and (b) the formation of a simple 1:1 complex ML through the equilibrium depicted on Equation (1). The latest assumption is generally valid for the polyamino carboxylic acids used in soil remediation.

(1) $$\begin{array}{l} \text{M}+\text{L}⇆\text{ML} \end{array}$$

The related complex formation constant is thus defined as:

(2) $$\begin{array}{l} K=\;\frac{\left[\text{ML}\right]}{\left({\text{L}}_{0}-\left[\text{ML}\right]\;\right){\left({\text{M}}_{0}-\left[\text{ML}\right]\right)}_{\;}} \end{array}$$

where M_0_ and L_0_ are the total concentration of metal and ligand, respectively. Equation (2) can then be rearranged in a suitable form in order to calculate the value of the constant *K* necessary to reach a desired amount of M_0_ in the complexed form (ML), expressed as the ratio of the complex concentration to the total concentration of metal, i.e., as *f* = [ML]/[M_0_], for given values of the total metal concentration M_0_ and of total ligand concentration L_0_ expressed by the ratio R = L_0_/M_0_. Equation (2) is thus transformed in Equation (3):

(3) $$\begin{array}{l} K=\;\frac{f}{{\text{M}}_{0}\left(\text{R}-f\;\right){\left(1-f\right)}_{\;}} \end{array}$$

If we have an estimate of the content of the metal ion in the soil, roughly assuming that it is totally transferred to solution when treating a given weight of soil with a defined volume of a solution of the chelating agent with a L_0_ concentration, we can establish the values M_0_ and R. For clarification, in Table 2 we report the values of log *K* to obtain 25, 50, 75, and 99% of the total metal transformed in the complex form (i.e., *f* = 0.25, 0.50, 0.75, and 0.99 respectively) for different M_0_ concentration ranging from 2 × 10^−5^ M to 2 × 10^−3^ M, and for *R*-values from 2 to 100.

**Table 2:** Values of log *K* to obtain the values of *f* (ratio between complexed and total metal ion) reported in the first column, for given values of total metal ion M_0_ and ratio *R* = L_0_/M_0_.

		**log** ***K***			
**M_**0**_**	***f***	***R* = 2**	***R* = 10**	***R* = 50**	***R* = 100**
2 × 10^−5^ M	0.25	3.98	3.23	2.53	2.22
	0.50	4.52	3.72	3.00	2.70
	0.75	5.08	4.21	3.48	3.18
	0.99	6.69	5.74	5.00	4.70
2 × 10^−4^ M	0.25	2.98	2.23	1.53	1.22
	0.50	3.52	2.72	2.00	1.70
	0.75	4.08	3.21	2.48	2.18
	0.99	5.69	4.74	4.00	3.70
2 × 10^−3^ M	0.25	1.98	1.23	0.53	0.22
	0.50	2.52	1.72	1.00	0.70
	0.75	3.08	2.21	1.48	1.18
	0.99	4.69	3.74	3.00	2.70

For a better understanding of *Nurchi's method* and its way of application we will use, as examples several amino carboxylic ligands (see Table 3), largely used in soil remediation mainly due to their biodegradability properties. EDTA will be also considered for comparison purposes. The present ligands are characterized by a definite number *n* of protonation equilibria and, consequently, protons compete with metal ions for the same basic sites where protonation and metal coordination occur. In this case, the *effective* or *conditional stability constant* β_eff_ (Ringbom, 1963) is an actual measure of the real capacity of a ligand to bind a metal ion at any given pH value.

**Table 3:** Structure, IUPAC name, acronym and molecular formula of the ligands considered in this study.

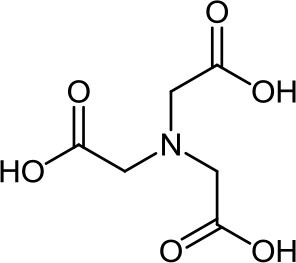	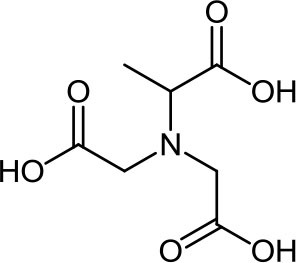	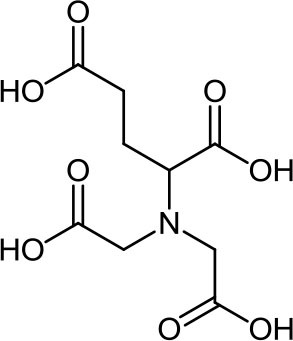
2, 2', 2” -Nitrilotriacetic acid **NTA**, C_6_H_9_NO_6_	*N, N*-Bis(carboxymethyl)alanine **MNTA**, C_7_H_11_NO_6_	*N, N*-Bis(carboxymethyl)-glutamic acid **GLDA**, C_9_H_13_NO_8_
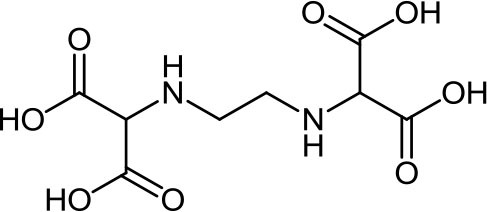	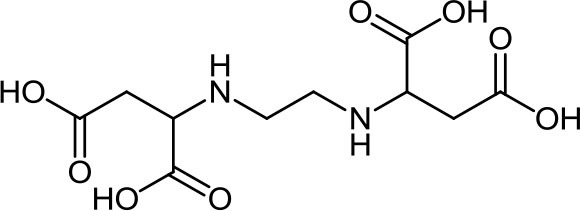	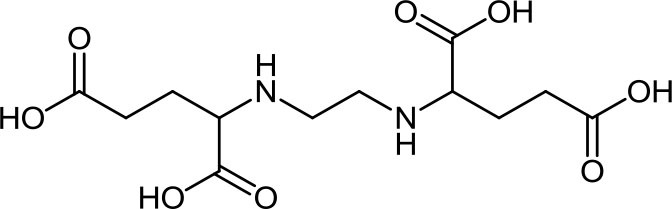
2, 2' -(1, 2-Ethanediyldiimino) dimalonic acid **EDDM**, C_8_H_12_N_2_O_8_	2, 2' -(1, 2-Ethanediyldiimino) disuccinic acid **EDDS**, C_10_H_16_N_2_O_8_	2, 2' -(1,2-Ethanediyldiimino) diglutamic acid **EDDG**, C_12_H_20_N_2_O_8_
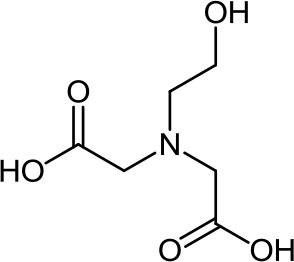	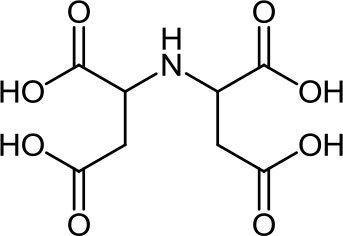	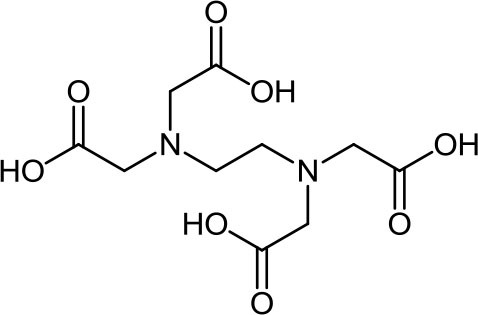
2, 2' -[(2-Hydroxyethyl) imino]diacetic acid **HIMDA**, C_6_H_11_NO_5_	2, 2' -Iminodisuccinic acid **IDS**, C_8_H_11_NO_8_	2, 2', 2”, 2”' -(1, 2-Ethanediyldinitrilo) tetraacetic acid **EDTA**, C_10_H_16_N_2_O_8_

The *effective stability constant*, expressed as a function of the total concentrations of chelating agent and metal ion, takes into account all the different protonated species according to the protonation equilibria summarized on Equation (4), and the complex formation equilibrium (Equation 5):

(4) $$\begin{array}{l} \;{\text{L}}^{\text{n−}}+\;{\text{H}}^{+}\;⇆\;{\text{LH}}^{\left(\text{n−1}\right)-}\\ {\text{LH}}^{\left(\text{n−1}\right)-}+\;{\text{H}}^{+}\;⇆\;{\text{LH}}_{2}^{\left(\text{n−2}\right)-}\\ \;...............................................\\ \;{\text{LH}}_{\left(\text{n−1}\right)}^{-}+\;{\text{H}}^{+}\;⇆\;{\text{LH}}_{\text{n}} \end{array}$$

(5) $$\begin{array}{l} {\text{L}}^{\text{n−}}+\;{\text{M}}^{\text{p+}}\;⇆\;{\text{LM}}^{\left(\text{n−p}\right)-} \end{array}$$

Being the complex formation constant described as:

(6) $$\begin{array}{l} \beta_{LM}=\;\frac{\left[{\text{LM}}^{\left(\text{n−p}\right)-}\right]}{\left[{\text{L}}^{\text{n−}}\right][{\text{M}}^{\text{p+}}]} \end{array}$$

The mass balance equation for total ligand can then be expressed by Equation (7):

(7) $$\begin{array}{l} {}[{\text{L}}_{\text{tot}}]=[{\text{L}}^{\text{n−}}]+[{\text{LH}}^{\left(\text{n−1}\right)-}]+[{\text{LH}}_{2}^{\left(\text{n−2}\right)-}]+...+[{\text{LH}}_{\text{n}}]\;\\ \;+[{\text{LM}}^{\left(\text{n−p}\right)-}] \end{array}$$

that, using the protonation constants of equilibria (4), is converted in:

(8) $$\begin{array}{l} \left[{\text{L}}_{\text{tot}}]=[{\text{L}}^{\text{n}-}](1+\beta_{1}[\text{H}]+\beta_{2}{\left[\text{H}\right]}^{2}+\beta_{3}{[\text{H]}}^{3}++\beta_{\text{n}}{[\text{H]}}^{\text{n}}\right)\\ \;+\;[{\text{LM}}^{(\text{n}-\text{p})-}] \end{array}$$

At this point, the complex formation constant [Equation (6)] can be written as:

(9) $$\begin{array}{l} \beta_{\text{LM}}=\;\frac{\left[{\text{LM}}^{\left(\text{n−p}\right)-}\right]}{\frac{\left(\left[{\text{L}}_{\text{tot}}\right]-\left[{\text{LM}}^{\left(\text{n−p}\right)-}\right]\right)}{\text{D}\left(\text{H}\right)}(\left[{\text{M}}_{\text{tot}}\right]-[{\text{LM}}^{\left(\text{n−p}\right)-}]\;)} \end{array}$$

and the effective stability constant β_*eff*_ can be determined as:

(10) $$\begin{array}{l} \beta_{\text{eff}}=\frac{\beta_{\text{LM}}}{\text{D}\left(\text{H}\right)}\\ \;=\;\frac{\left[{\text{LM}}^{\left(\text{n−p}\right)-}\right]}{\left(\left[{\text{L}}_{\text{tot}}\right]-\left[{\text{LM}}^{\left(\text{n−p}\right)-}\right]\right)(\left[{\text{M}}_{\text{tot}}\right]-[{\text{LM}}^{\left(\text{n−p}\right)-}]\;)} \end{array}$$

Interestingly, Equation (10) is formally identical to Equation (2). As such, all the considerations made above, regarding the values of log *K* necessary to reach a given percent of the total metal transformed in the complex form (*f* = 0.25, 0.50, 0.75, and 0.99 respectively) for different M_0_ values, are valid, as well as the values reported in Table 2.

**Figure 1:**
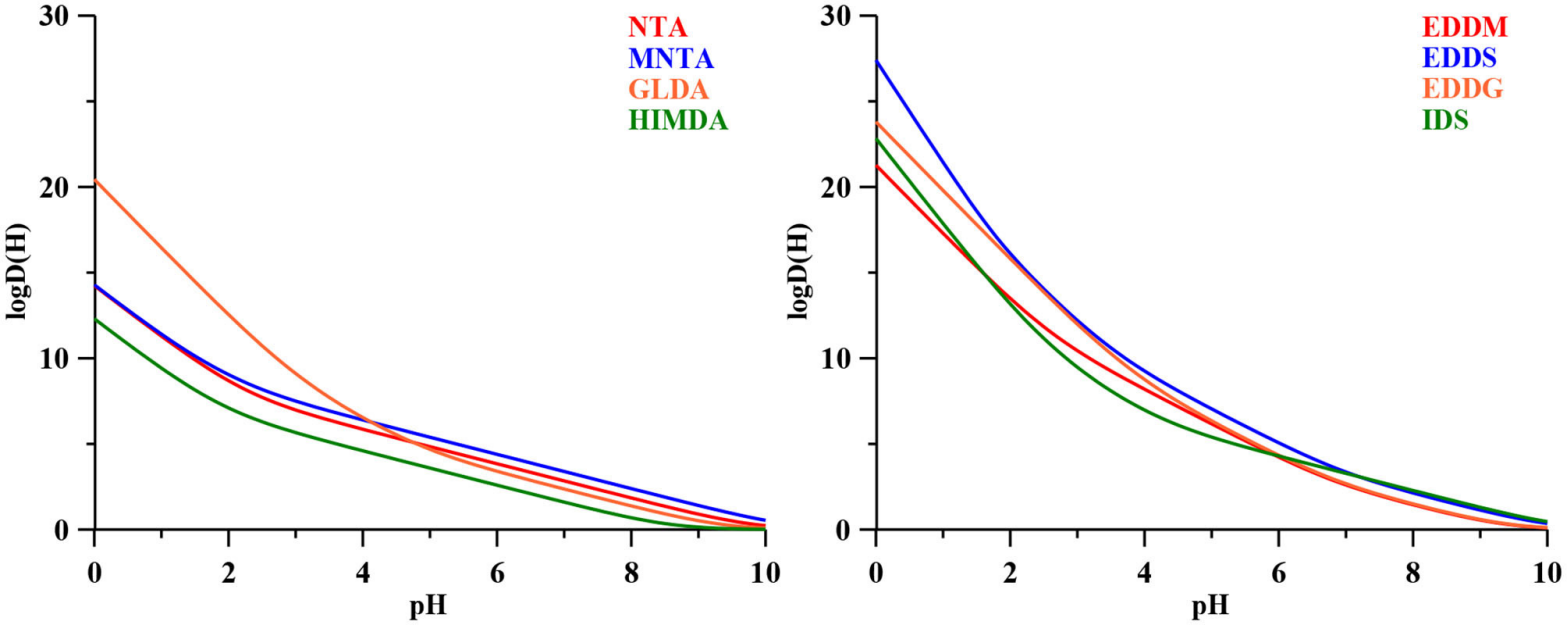
The log *D(H)* functions for the amino carboxylic ligands considered in this study (Table 3), calculated with the literature protonation constants presented in Table 4.

With base on the literature protonation constants reported in Table 4, we could calculate the representative curves of log *D(H)* values as a function of pH for all the eight ligands in study. The corresponding trend lines are presented in Figure 1. In Table 4 are also depicted the complex formation constants for the mentioned ligand toward several metal ions of interest.

**Table 4:** Protonation and complex formation constants of the amino carboxylic ligands considered in this study.

	**log** *****β*****								
**Species**	**NTA**	**MNTA^**9**^**	**GLDA^**10**^**	**HIMDA**	**EDDM**	**EDDS**	**EDDG**	**IDS**	**EDTA**
LH	9.84^1^	10.39	9.36	8.59^11^	9.4^16^	10.1^20^	9.46^25^	10.28^29^	10.21^34^
LH_2_	12.40^1^	12.83	14.39	10.83^11^	16.16^16^	17.01^20^	16.27^25^	14.77^29^	16.41^34^
LH_3_	14.23^1^	14.27	17.88	12.29^11^	19.06^16^	20.85^20^	20.52^25^	18.23^29^	19.18^34^
LH_4_			20.44		21.27^16^	23.90^20^	23.80^25^	20.81^29^	21.20^34^
LH_5_						25.3^20^		22.83^29^	
LH_6_						27.4^20^			
CdL	9.8^2^	10.61		7.21^12^		10.3^21^	8.76^25^		16.41^35^
PbL	11.34^3^	12.07		9.45^3^	11.12^17^	11.3^21^	8.62^25^		17.93^35^
HgL	13.48^4^		14.33	5.48^7^	18.64^16^	14.40^22^	16.66^25^		22.02^36^
FeL	15.87^5^			11.60^11^		20.6^20^	15.7^26^	14.70^30^	25.10^37^
MnL	7.44^6^			5.55^7^	8.50^18^	8.63^22^	6.74^27^		13.8^35^
CuL	12.94^3^	13.88	13.09	11.72^3^	13.0^19^	18.7^20^	12.65^28^	12.69^31^	18.7^35^
ZnL	10.67^7^	11.06		8.02^13^		13.58^20^	10.25^25^	10.30^32^	16.39^35^
CaL	6.57^8^	6.97	5.18	4.58^14^	4.80^16^	4.58^23^	2.59^25^	4.63^33^	10.69^35^
MgL		5.83	5.93	3.5^15^	4.51^16^	5.82^24^	3.0^25^	5.52^33^	8.9^35^

1 *Ref. Daniele et al. (1985), I = 0.1 M (R4N.X) and T = 25°C*.

2 Ref. Schwarzenbach and Gut (1956), I = 0.1 M (KNO_3_) and T = 20°C.

3 Ref. Felcman and Da Silva (1983), I = 0.1 M (KNO_3_) and T = 25°C.

4 Ref. Gritmon et al. (1977), I = 0.5 M (NaClO_4_) and T = 25°C.

5 Ref. Motekaitis and Martell (1994), I = 0.1 M (KCl) and T = 25°C.

6 Ref. Schwarzenbach and Freitag (1951), I = 0.1 M (KCl) and T = 20°C.

7 Ref. Schwarzenbach et al. (1955), I = 0.1 M (KCl) and T = 20°C.

8 Ref. Moeller and Ferrus (1962), I = 0.1 M (KNO_3_) and T = 25°C.

9 Ref. Riečanská et al. (1974), I = 0.1 M (KNO_3_) and T = 20°C.

10 Ref. Gorelov and Nikolskii (1977), I = 0.1 M (KNO_3_) and T = 25°C.

11 Ref. Anderegg, 1986, I = 1.0 M (NaClO_4_) and T = 25°C.

12 Ref. Kodama et al. (1974), I = 0.3 M (NaNO_3_) and T = 25°C.

13 Ref. Kodama (1974), I = 0.3 M (NaClO_4_) and T = 25°C.

14 Ref. Mighri and Rumpf (1975), I = 0.1 M (?) and T = 25°C.

15 Ref. Verdier and Piro (1969), I = 0.1 M (NaClO_4_) and T = 25°C.

16 Ref. Gorelov and Babich (1972b), I = 0.1 M (KNO_3_) and T = 25°C.

17 Ref. Gorelov et al. (1973), I = 0.1 M (KNO_3_) and T = 25°C.

18 Ref. Samsonov and Gorelov (1974), I = 0.1 M (KNO_3_) and T = 25°C.

19 Ref. Mashihara et al. (1973), I = 0.1 M (KNO_3_) and T = 25°C.

20 Ref. Orama et al. (2002), I = 0.1 M (NaCl) and T = 25°C.

21 Ref. Vasilev et al. (1990), I = 0.1 M (KNO_3_) and T = 25°C.

22 Ref. Vasilev and Zaitseva (1993), I = 0.1 M (KNO_3_) and T = 25°C.

23 Ref. Vasilev et al. (1989), I = 0.1 M (KNO_3_) and T = 25°C.

24 Ref. Gorelov and Babich (1971), I = 0.1 M (KNO_3_) and T = 25°C.

25 Ref. Gorelov and Babich (1972a), I = 0.1 M (KNO_3_) and T = 25°C.

26 Ref. Sunar and Trivedi (1971), I = 0.1 M (KNO_3_) and T = 30°C.

27 Ref. Samsonov and Gorelov (1974), I = 0.1 M (KNO_3_) and T = 25°C.

28 Ref. Trivedi et al. (1971), I = 0.1 M (KNO_3_) and T = ?.

29 Ref. Knyazeva et al. (2002), I = 0.1 M (KCl) and T = 25°C.

30 Ref. Nikol'skii and Knyazeva (2002), I = 0.1 M (KCl) and T = 25°C.

31 Ref. Vasilev and Al (1998), I = 0.1 M (KNO_3_) and T = 25°C.

32 Ref. Vasilev and Al (1994), I = 0.1 M (?) and T = 25°C.

33 Ref. Vasilev et al. (1999), I = 0.1 M (KNO_3_) and T = 25°C.

34 Ref. Gridchin et al. (2004), I = 0.1 M (KNO_3_) and T = 25°C.

35 Ref. Sarma and Ray (1956), I = 0.1 M (NaClO_4_) and T = 25°C.

36 Ref. Brunetti et al. (1969), I = 0.1 M (KNO_3_) and T = 25°C.

37 *Ref. Delgado et al. (1997), I = 0.1 M (KNO_3_) and T = 25°C*.

These functions constitute the basis for determining the behavior of each ligand. In fact, if we present Equation (10) in its logarithmic form (Equation 11) we can see that function *F(H)* is obtained by adding to log *D(H)* the log βeff required for the transformation of the given % of total metal ion in the complexed form.

(11) $$\begin{array}{l} F(H)=\log\beta_{\text{eff}}+\log D(H)=\log\beta_{\text{LM}} \end{array}$$

**Figure 2:**
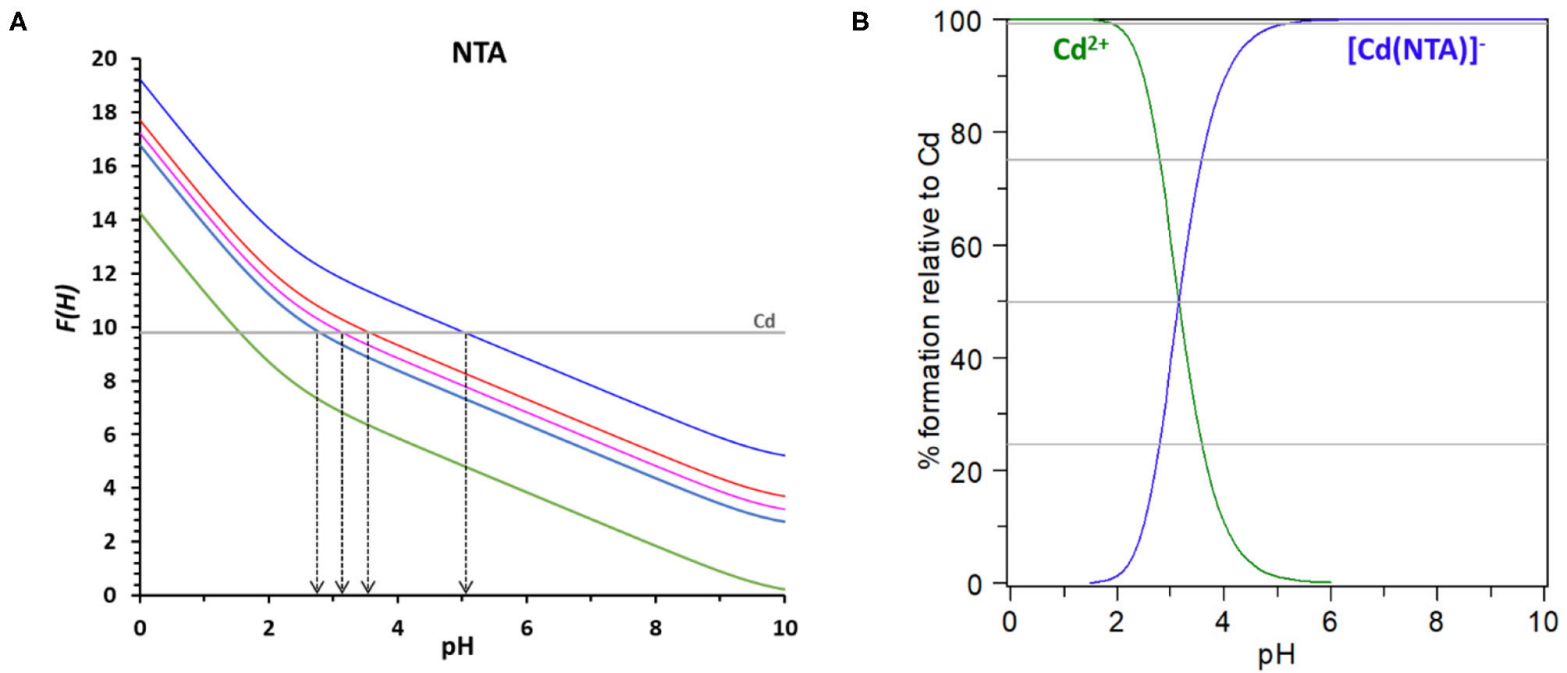
**(A)** The *D(H)* function for NTA is reported in red, the *F(H)* functions for the system Cd-NTA with total cadmium 2 × 10^−5^ M and *R* = 50 are reported for the 0% of complex formation (green), 25% (light blue), 50% (pink), 75% (red) and 99% (blue). **(B)** Speciation plot for the system Cd-NTA with [Cd_tot_] = 2 × 10^−5^ M and [NTA_tot_] = 1 × 10^−3^ M (*R* = 50).

*F(H)* representation will then be the basis for the evaluation of the chelating ability of the ligands of interest on *Nurchi's method*. For a clear elucidation of the method, we will now describe, in detail, the procedure applied to the system NTA/Cd^2+^, with M_0_ = 2 × 10^−5^ *M* and *R* = 50. In such a case, the value of log β_eff_ to reach 25% of complexed Cd^2+^ is 2.53, for 50% is 3.00, for 75% is 3.48 and is 5.00 for 99% (Table 1). These values, added to the function *D(H)* for NTA reported in green in Figure 2A, allow the calculation of the *F(H)* functions for NTA corresponding to 25, 50, 75, and 99 % of complex formation reported in different colors in the same Figure 2A. The value log β_CdNTA_ = 9.8 is reported as a straight line parallel to the pH axis. This line intersects the 25% *F(H)* function at pH = 2.79, the 50% *F(H)* function at pH = 3.15, the 75% *F(H)* function at pH = 3.58 and the 99% *F(H)* function at pH = 5.05. It means that the log β_CdNTA_ = 9.8 determines a conditional constant at pH = 2.79 that allows the 25% complexation of total cadmium, and so on till the 99% of complexation at pH = 5.05.

The same information can be obtained from the speciation plot, calculated with Hyss program (Alderighi et al., 1999) for the referred system (Figure 2B), where can be easily seen that the formation curve of the cadmium complex reaches 25% at pH = 2.79, 50% at pH = 3.15, 75% at pH = 3.87 and 99% at pH = 5.05.

**Figure 3:**
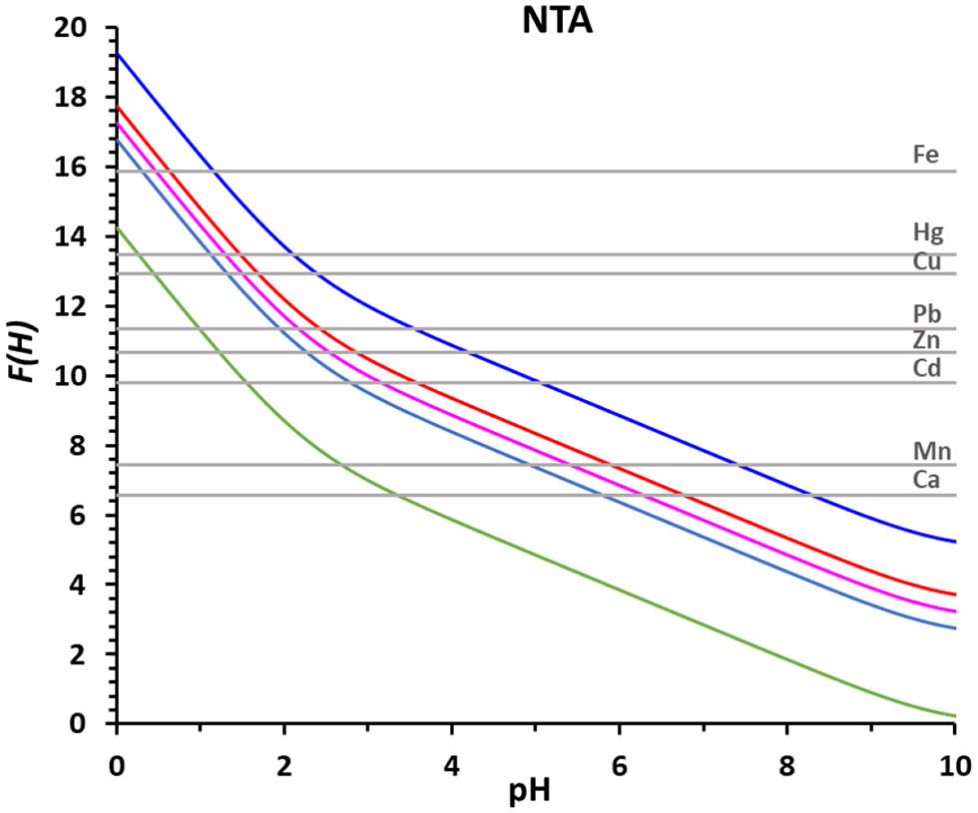
The *F(H)* functions of NTA (total metal ion concentration 2 × 10^−5^ M and *R* = 50) for 0% of complex formation (green), 25% (light blue), 50% (pink), 75% (red), and 99% (blue). The straight gray lines correspond to the log β_LM_ of the indicated metal ions with NTA.

The advantage of the proposed method based on the *F(H)* function is most evident in Figure 3 were the log β_MNTA_'s of NTA with all the metal ions reported in Table 3 are represented as straight gray lines. Figure 3 gives at a glance the pH ranges at which the complexation of each metal ion takes place, that could be deducted in a very laborious way from the corresponding speciation plots created for all the metal ions with NTA, HIMDA, EDDG, EDDS, and EDTA, from the stability constants reported in Table 4 (summary in Supplementary Figure 1).

It is clearly inferred, from Figure 3, that Fe^3+^, the metal ion with the strongest stability constant (log β_LM_ = 15.87), is already complexed at low pH values (25% at pH 0.3 and 99% at pH 1.2) and the complex formation happens in a narrow range (0.9 pH units from 25 to 99%). On the contrary, Ca^2+^, the metal ion with the lowest stability constant (log β_LM_ = 6.57), is complexed at higher pH values (25% at pH 5.8 and 99% at pH 8.3) and the complex formation happens in a broader pH range (2.5 pH units from 25 to 99%).

The range of complexation increases as the complexation occurs in a flatter part of curve *D(H)*, and so of curve *F(H)*. As mentioned above, once calculated the *D(H)* curve for a given ligand, the *F(H)* curves at different M_0_ and R values can be easily derived by adding to the *D(H)* values the proper values of log β_eff_.

**Figure 4:**
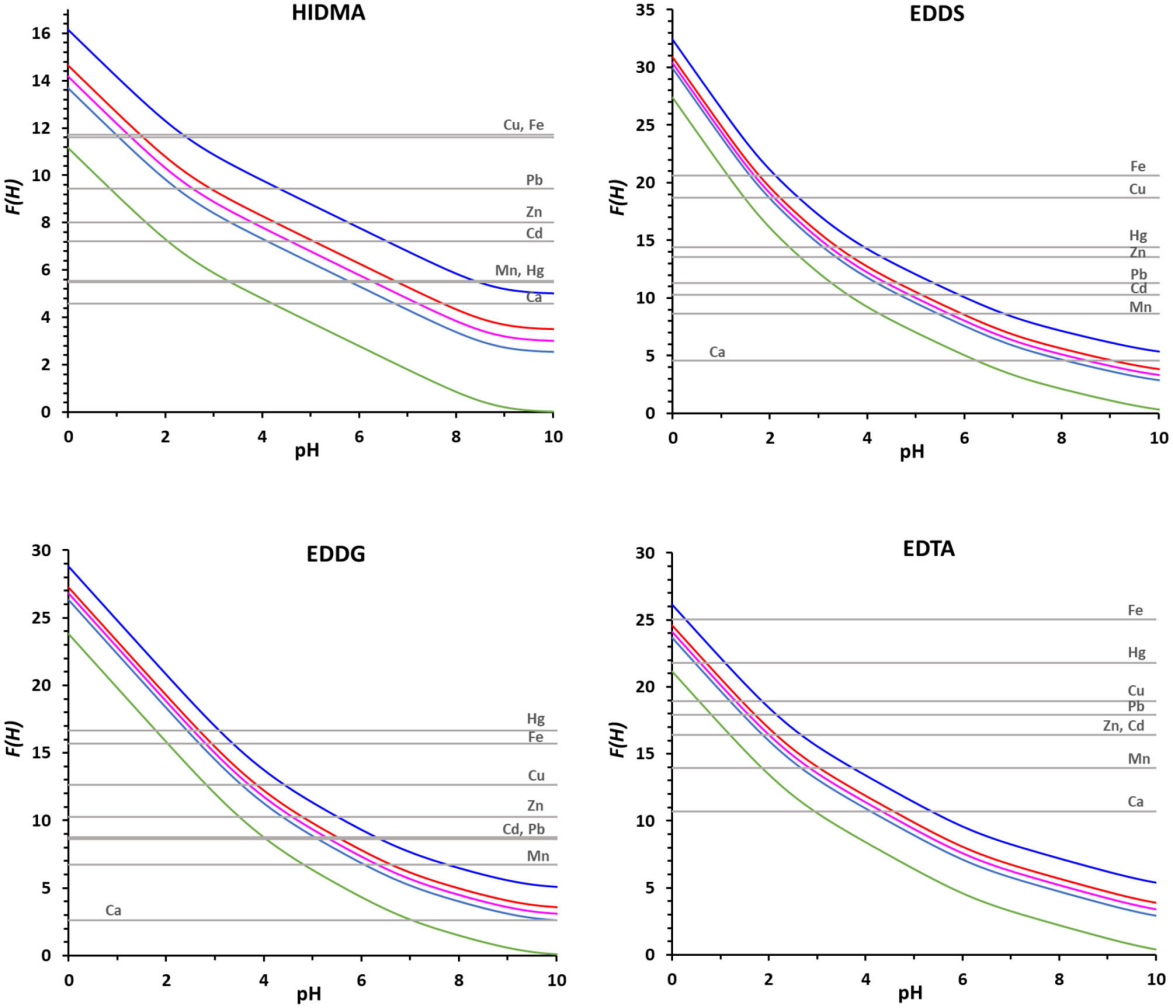
The *F(H)* functions for the ligands HIMDA, EDDS, EDDG and EDTA with the metal ions in Table 3, at [M_tot_] 2 × 10^−5^ M and *R* = 50, are reported for the 0% of complex formation (green), 25% (light blue), 50% (pink), 75% (red) and 99% (blue).

Using the proposed method and with base on the complex formation constants reported in Table 4, we could compare the binding ability of the four ligands for which literature data are available for all the chosen metal ions (NTA, HIMDA, EDDS, EDDG), and the corresponding *F(H)* plots are presented in Figure 4. For simplifying the discussion, we collected from these plots the pH range of complex formation for the eight metal ions and the four ligands, i.e., the pH of the intersection points of log β_LM_ with the *F(H)*_25%_ and the *F(H)*_99%_ (Table 5). Lower intervals correspond to a stronger complex. As a first step, a comparison can be made between the structurally similar NTA and HIMDA: the lack of the third carboxylic group in HIMDA drastically reduces its binding ability, in a marked extent with Hg^2+^ and in a minor extent in Mn^2+^, Cd^2+^, Zn^2+^ and Fe^3+^, being Cu^2+^ practically unaffected. In case of Cu^2+^ we hypothesize that this is due to the fact that this metal ion may form a stable square planar complex with HIMDA, structurally similar to that with Pd^2+^ (BIACDD structure, Supplementary Figure 3). The comparison between EDDS and EDDG shows a slightly higher coordination capability of EDDS for all metal ions, except for Hg^2+^. Presumably, the longer arms connecting two of the carboxylic groups favor the coordination of the larger mercuric ion.

**Table 5:** pH range of complex formation (from 25 to 99%) for different metal ions with the indicated chelating agents.

	**pH range**				
**Metal ion**	**NTA**	**HIMDA**	**EDDG**	**EDDS**	**EDTA**
Fe^3+^	0.30–1.15	1.02–2.46	2.69–3.40	1.56–2.13	<0–0.3
Hg^2+^	1.12–2.06	5.81–8.51	2.41–3.12	3.12–3.93	0.4–1.1
Cu^2+^	1.28–2.32	0.99–2.41	3.55–4.45	1.99–2.59	1.2–1.9
Pb^2+^	1.84–3.55	2.27–4.40	5.05–6.38	4.18–5.34	1.5–2.2
Zn^2+^	2.27–4.20	3.35–5.82	4.40–5.60	3.41–4.26	1.9–2.7
Cd^2+^	2.70–5.00	4.16–6.67	5.05–6.38	4.68–5.86	1.9–2.7
Mn^2+^	4.85–7.45	5.81–8.51	6.05–7.80	5.46–6.81	2.7–3.8
Ca^2+^	5.80–8.27	6.75–> 10.0	9.93–> 10.0	8.08–> 10.0	4.1–5.4

EDTA is also reported, as being one of the most used chelating agents. From the plots presented in Figure 4, and data on Table 5, we can see that EDTA is, among all the evaluated ligands, the most efficient chelator for all the metals considered. Although, in case of Cu^2+^ and Hg^2+^, NTA can be used with the same expected rate of success in terms of metal remediation.

This preliminary screening gives evidence of the superiority of EDTA, immediately followed by NTA among the examined ligands, and of the inability of HIMDA to act as a ligand for environmental remediation. EDDS, and in a lower extent, EDDG can instead be used, even if with a lower capability.

We must remember that the results here reported assumed the presence of a free metal ion in the soil in contact with the washing solution. In reality, the metal ion is usually complexed by the inorganic and organic components of the soil, and the complex formation reaction is, in reality, a competition between the chelating agent and the soil ligands for the target metal ion. Since the hydroxide formation constitutes probably the most competitive reaction, we checked the validity, or the limits, of our assumptions recalculating the speciation plots (Supplementary Figure 2) this time using a model that included the hydroxide formation constants [Supplementary Table 1, from Baes and Mesmer (Baes and Mesmer, 1976)]. As can be observed, in the generality of the cases, the inclusion of hydrolysis equilibria does not change the speciation plots. Only in the case of Hg^2+^ and Fe^3+^ the formation of hydroxides is apparent at high pH values. In particular, the formation of the weak complex between HIMDA and Hg^2+^ is completely hindered, and partially the formation of the complex with EDDS. In this way we can conclude that the action of neglecting the competitive reactions of hydrolysis can be considered valid in the majority of situations. Furthermore, HIMDA can form with Fe^3+^ complexes with different protonation degrees, instead of the unique assumed complex, but this ligand presents peculiar features that differentiate it from amino poly-carboxylic ligands.

To sum up, we can say that *Nurchi's method* provides a primary fundamental picture of the main features of the complexing ability of a given chelator as the minimum pH values at which it can operate and the competition among the present metal ions, but most importantly permits a preliminary screening between a set of potential ligands. This treatment can act as a guide in the design of metal chelators specific for target metal ions by showing the behavior of existing ligands characterized by selected binding groups, as, for example, mercapto ligands for mercury.

## Conclusion

The method here proposed allows determining both the suitability of the ligand for scavenging the target metal ions, and the minimal concentration of ligand to be used, minimizing the adverse effects on essential metal ions. This is particularly relevant when studying the real application of a washing ligand to a specific soil, knowing the total concentrations of metals to be removed before the experimental determination of its behavior. For that purpose, the use of databases of stability constants is highly recommended, together with speciation programs.

We would remember here that different approaches have been proposed in the literature to assess the sequestering ability of ligands toward metal cations, which can be complementary to the here proposed method. The reviews by Bazzicalupi et al. (2012) and by Crea et al. (2014) examine these treatments and thoroughly discusses their *pros* and *cons*. The necessity to use suitable data, obtained in the possible similar conditions to those of soils under treatment and the necessity of reliable stability constants must be stressed. In essence, inefficient treatments and severe procedural errors can sometimes derive from the use of bad/unreliable data. Besides allowing a preliminary screening of the binding ability of chelating agents toward the target polluting metal ions, and those typical of the soils, the proposed method can be applied in the multiple uses of chelating agents, spanning from biomedical to industrial applications. As an example, we can envisage its use on the evaluation of the role of different biomolecules, as for example bacterial metallophores, on metal uptake and homeostasis in living organisms. This information is paramount for different actions, namely, for the development of new strategies for the fight of antibiotic resistant pathogenic bacteria.

## Author Contributions

VN, GC, and RB: conceptualization. RC, GS, and SG: data elaboration. VN, GC, SG, and RB: data curation. All authors have read and agreed to the published version of the manuscript.

## Conflict of Interest

The authors declare that the research was conducted in the absence of any commercial or financial relationships that could be construed as a potential conflict of interest.
