# Supplementary material for: Chelating Agents in Soil Remediation: A New Method for a Pragmatic Choice of the Right Chelator
Source: Front Chem. 2020 Nov 2;8:597400. doi: 10.3389/fchem.2020.597400 (PMC7667266; doi:10.3389/fchem.2020.597400)
Supplement: Supplementary file 1 [file Data_Sheet_1.docx]

Chelating agents in soil remediation:

A new method for a pragmatic choice of the right chelator.

Valeria Marina Nurchi^1*^, Rosita Cappai^1^, Guido Crisponi^1^, Gavino Sanna^2^, Giancarla Alberti^3^, Raffaela Biesuz^3^, Sofia Gama^4^

^1^Dipartimento di Scienza della Vita e dell’Ambiente, University of Cagliari, Cittadella Universitaria, 09042 Monserrato, Italy

^2^Dipartimento di Chimica e Farmacia, University of Sassari, Via Vienna 2, 07100 Sassari, Italy

^3^Dipartimento di Chimica, University of Pavia, Via Taramelli 12, 27100 Pavia, Italy.

^4^Department of Analytical Chemistry, Faculty of Chemistry, University of Białystok, ul. Ciołkowskiego 1K, 15-245 Białystok, Poland

*** Correspondence:** Valeria Marina Nurchi [nurchi@unica.it](mailto:nurchi@unica.it)

Keywords: Chelating agents, speciation, soil remediation, metal pollution

**Supplementary Information**

|  |  |  |
| --- | --- | --- |
|  |  |  |
|  |  |  |
|  |  |  |
|  |  |  |
|  |  |  |
|  |  |  |
|  |  |  |
|  |  |  |
| **** | **** | **** |
| **** | **** | **** |
| **** | **** |  |
|  |  |  |
|  |  |  |
|  |  |  |

**Fig. 1S.** Speciation plots for the systems M-NTA, M-HIMDA, M-EDDG, M-EDDS and M-EDTA with M = Cd^2+^, Pd^2+^, Hg^2+^, Fe^3+^, Mn^2+^, Cu^2+^, Zn^2+^ and Ca^2+^, [M_tot_] = 2×10^−5^ M and [L_tot_] = 1×10^−3^ M (R = 50).

| **** | **** | **** |
| --- | --- | --- |
| **** | **** | **** |
| **** |  |  |
| **** | **** | **** |
| **** | **** | **** |
| **** |  |  |
| **** | **** | **** |
| **** | **** | **** |
| **** |  |  |
| **** | **** | **** |
| **** | **** | **** |
| **** |  |  |

**Fig. 2S.** Speciation plots for the systems M-NTA, M-HIMDA, M-EDDG and M-EDDS with M = Cd^2+^, Pd^2+^, Hg^2+^, Fe^3+^, Mn^2+^, Cu^2+^ and Zn^2+^, [M_tot_] = 2×10^−5^ M and [L_tot_] = 1×10^−3^ M (R = 50), taking into account in HYSS calculations the hydrolysis equilibria of referred metal ions (the used hydrolysis constants are reported in Table 1S).


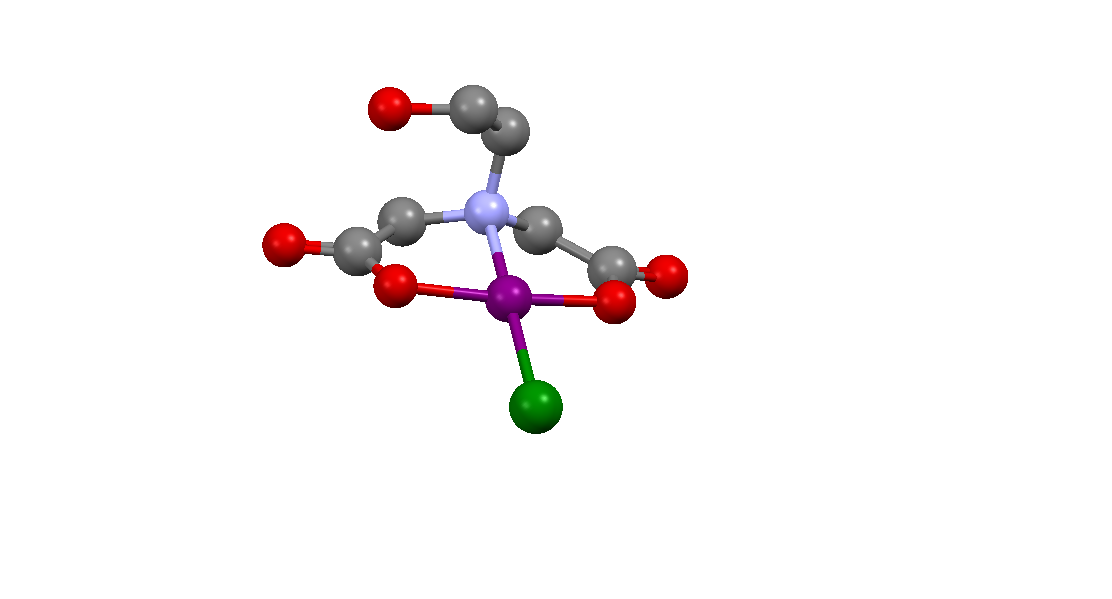


**Fig. 3S.** Cristal structure of the [Pd(HIMDA)Br] complex. ([I.N.Polyakova et al., 1981](#_ENREF_2)) Carbon in grey, nitrogen in violet, oxygen in red, bromide in green and metal ion in purple. Hydrogen atoms are omitted for simplification. Coordinates obtained from the Cambridge Structural Database, images created with Mercury 3.5.

**Table 1S.** Hydrolysis constants (log *β*) of Fe^3+^, Hg^2+^, Cu^2+^, Pb^2+^, Zn^2+^, Cd^2+^, Mn^2+^ and Ca^2+^ used in HYSS calculations. Values at 25 °C and 0.1 M ionic strength, from Baes and Mesmer ([Baes and Mesmer, 1976](#_ENREF_1)).

| **Fe^3+^** | | **Hg^2+^** | | **Cu^2+^** | | **Pb^2+^** | |
| --- | --- | --- | --- | --- | --- | --- | --- |
| FeOH^2+^ | -2.563 | HgOH | -3.595 | CuOH^+^ | -8.220 | PbOH | -7.846 |
| Fe(OH)_2_^+^ | -6.205 | Hg(OH)_2_ | -6.336 | Cu(OH)_2_ | -17.525 | Pb(OH)_2_ | -17.244 |
| Fe(OH)_3_ | -12.497 | Hg(OH)_3_ | -21.100 | Cu(OH)_3_^-^ | -27.804 | Pb(OH)_3_ | -27.969 |
| Fe(OH)_4_^-^ | -21.883 | Hg_2_(OH) | -3.084 | Cu(OH)_4_^2-^ | -39.125 | Pb_2_(OH) | -6.176 |
| Fe_2_(OH)_2_^4+^ | -2.843 | Hg_3_(OH)_3_ | -6.420 | Cu_2_(OH)_2_^2+^ | -10.598 | Pb_3_(OH)_4_ | -23.882 |
| Fe_3_(OH)_4_^5+^ | -6.054 |  |  |  |  | Pb_4_(OH)_4_ | -20.288 |
|  |  |  |  |  |  | Pb_6_(OH)_8_ | -43.236 |

| **Zn^2+^** | | **Cd^2+^** | | **Mn^2+^** | | **Ca^2+^** | |
| --- | --- | --- | --- | --- | --- | --- | --- |
| ZnOH | -9.145 | CdOH | -10.308 | MnOH | -10.794 | CaOH | -12.850 |
| Zn(OH)_2_ | -17.095 | Cd(OH)_2_ | -20.586 | Mn(OH)_2_ | -22.416 |  |  |
| Zn(OH)_3_^-^ | -28.390 | Cd(OH)_3_ | -33.300 | Mn(OH)_3_ | -34.805 |  |  |
| Zn(OH)_4_^2-^ | -40.709 | Cd(OH)_4_ | -46.911 | Mn(OH)_4_ | -47.843 |  |  |
| Zn_2_(OH)^3+^ | -8.894 | Cd_2_(OH) | -9.155 | Mn_2_(OH) | -10.315 |  |  |
| Zn_2_(OH)_6_^2-^ | -57.534 | Cd_4_(OH)_4_ | -32.364 | Mn_2_(OH)_3_ | -24.391 |  |  |

**References**

Baes, C.F., and Mesmer, R.E. (1976). *The Hydrolysis of Cations.* New York: John Wiley & Sons.

I.N.Polyakova, T.N.Polynova, and Porai-Koshits, M.A. (1981). *Koord. Khim.* 7**,** 1737.
